# The attitude and perceptions of doctors at Letaba Hospital towards family medicine: A qualitative study

**DOI:** 10.4102/safp.v62i1.5034

**Published:** 2020-07-27

**Authors:** Christian N. Saidiya, Gert J.O. Marincowitz, Doudou K. Nzaumvila, Tombo Bongongo, Indiran Govender

**Affiliations:** 1 Department of Family Medicine Sefako Makgatho Health Sciences University, Pretoria, South Africa; 2 Department of Health, Limpopo, South Africa; 3 Department of Family Medicine, University of Limpopo, South Africa; 4 Department of Family Medicine and Primary Health Care, Sefako Makgatho Health Sciences University, Pretoria, South Africa

**Keywords:** Doctors’ perceptions towards family medicine, community health services, primary healthcare professionals, doctors’ misconceptions of family medicine, family medicine

## Abstract

**Background:**

It was noted that family medicine (FM) was not used properly by doctors at Letaba Hospital. Anecdotally, misconceptions and misunderstandings about FM were reported. An exploration was recommended to understand the perceptions and attitudes of doctors at Letaba Hospital with regard to FM. Identifying doctors’ misconceptions and the possible reasons for mistaken beliefs about FM by other specialists could offer possible solutions.

**Methods:**

A qualitative study was conducted that attempted to identify the perceptions of doctors about FM and to explore their attitudes towards this specialty.

**Results:**

The primary findings indicate more positive than negative perceptions of other disciplines towards FM. The participants viewed FM as the centre of the healthcare system, with prevention being its core business. This includes a holistic approach, the continuity of care, being community-based, and receiving recognition as a specialty. Family medicine was described by various medical personnel as making a positive contribution to the healthcare system. They note the role of FM discipline in district hospitals, its support of primary health- care and its ability to fill the gaps in the healthcare system, including surgical skills. The few negative perceptions that were identified mostly related to the status of FM as a specialty and doctors’ level of surgical ability. Based on individual interviews, 11 themes were extracted and a ‘wheel’ model was created, depicting the core values of the FM discipline.

**Conclusion:**

The study concluded that most participants have a positive perception of the role of FM, similar to the views of the senior staff in the discipline itself. The concerns from most participants are in the area of preventative medicine, which has not been given enough priority in South Africa and where doctors are expected to rapidly attend to long queues and manage casualties. There was also concern of a perceived lack of surgical skills.

## Background

The practice of family medicine (FM) can be traced back to prehistoric times.^1^ According to the American Academy of Family Physicians and others, it is undoubtedly the oldest of all specialities.^1,2^ This is in contrast to the many years before FM was recognised as a speciality by different medical boards and local authorities.^1,3^ Such as in the United States, FM was recognised in 1969,^2^ whereas in South Africa (SA) it was recognised in 2007.^3^ This is due to its wide scope of practice, which has confused FM with general medicine for many years.^1,2^ Currently in SA, as a speciality FM has a clear postgraduate training programme in the nine medical schools.^4^ This postgraduate level has a 2-year diploma^5^ and a 4-year Master of Medicine degree.^6^

Family medicine dedicated its early years to the provision of comprehensive medical services to patients of all ages and backgrounds.^1,2,7^ Currently, more is expected of family physicians (FPs), such as a capacity builder, consultant, care provider, clinical trainer and supervisor, leader, and champion of clinical governance and community-oriented primary care.^7^ The current scope of practice of FM covers a great number of medical skills and competences, putting FPs at the centre of the universal health coverage and primary healthcare (PHC). Family physicians are part of a multidisciplinary team within a medical and social network, acknowledged by international health organisations.^8,9^ Recent research data, policy papers and public statements from global health leaders have endorsed the contribution of FM to the improvement of the health system.^9^

Although the impact and need of the FP is becoming increasingly recognised by South Africa’s health districts,^10,11,12^ the concept of FM does not enjoy recognition from other specialities in terms of substance. In other words, other specialities can be described by a single word (e.g. paediatrics for children, orthopaedics for bones, and so forth), but FM can only be described by its principles. In many developing and developed nations, the concept is frequently interchangeable with general medical practice. This has largely to do with the different roles of the FM discipline, a complex and diverse specialty, where it is not clear whether all doctors share a common view in terms of its core business. African leaders are convinced of the potential contribution of FM to the health- care systems in Africa. However, they are unclear about what exactly the role of the FPs is, or should be.^13^ Family physicians themselves feel that their function is not well understood among other medical specialities.^14,15^ In addition, they are not afforded respect from other medical disciplines.^16^ A qualitative systematic review which explored students’ perceptions and attitudes about FM in Australia, Canada, Japan, Malaysia, Spain and Great Britain found that students perceived FM to be a career of low interest and prestige.^17^ Essuman and colleagues reported the same findings in Ghana.^18^

At the time that the survey was conducted, little was known of how other medical disciplines viewed FM. This article attempts to describe how other disciplines see the role of an FP at Letaba Hospital. The work aimed at developing an understanding of the perceptions and attitudes of doctors about FM at the hospital. The other objectives were to identify doctors’ misconceptions and the possible reasons for it.

## Methods

An exploratory qualitative study was conducted between August and September 2013 at Letaba Hospital. It is a Level 2 hospital located in the Mopani district, 20 km from Tzaneen, the closest town. When the study was conducted, Letaba had about 250 active beds, serving as a referral hospital for the six district hospitals in the Mopani district, with a population of 1 118 933 people.^19^ About 81% of the population of the Mopani district is rural. Youth and young adults constitute 40% of the population (females 53%) and 43% of the population live in poverty.^20^ At the time we conducted this study, the medical staff comprised 18 interns, 28 medical officers and seven specialists.^21^

Purposeful sampling was used. The principal researcher selected participants capable of bringing significant information or who could offer the richest source of data based on their professional judgement. The criterion for inclusion was doctors working at Letaba Hospital for at least 3 months; all intern doctors were excluded and doctors working in the FM department were also excluded. The selected doctors were invited to take part in an interview and were reminded on the days when interviews would be conducted. However, for various reasons, very few arrived to be interviewed.

A trained independent interviewer was appointed to facilitate all interviews. Free attitude individual interviews were conducted with a single open-ended exploratory question: ‘What are the attitudes and perceptions of doctors at Letaba Hospital about the discipline of FM?’ It was intended explicitly for the group and indirectly for the individual to encourage the participants to open up without fear of voicing their opinions. The interviewer made use of the reflective summary technique and clarifications while the interviews continued, until no new data emerged. Saturation was reached at the seventh interview, indicating that no further new information was gathered. The sample size purposefully collected was made of seven doctors, among whom three were specialists and four were medical doctors. Each interview recording (audiotape) was transcribed verbatim, and field notes were taken. All verbatim transcripts were double-checked for accuracy by the researcher’s assistant and the lead researcher. Finally, for respondent validation, all the transcripts were sent back to the participants to ascertain that they were a true reflection of what had been expressed during the interview.

Through using Microsoft Word’s colour coding and cut and paste methods, the researchers were able to manually verify the data, categorise it into themes and examine the interrelation between different categories, themes and concepts. The final result was then represented in a model where, under each theme, sufficient evidence was provided regarding quotes from the raw data and the demographics of the participants.

### Ethical consideration

Ethical clearance was obtained from the University of Limpopo, Medunsa Campus Research and Ethics Committee (MREC) with reference MREC/M/03/2013: PG and from the Department of Health Limpopo (reference no. 4/2/2). All participants provided informed consent.

## Results

Four out of seven study participants had worked at Letaba Hospital for more than 5 years (see Table 1). This could have allowed them to form a critical view of FM, which contributed to the richness of the information.

**TABLE 1:** Baseline characteristics of respondents.

Participant	Age	Gender	Medical category and department	Years working as a specialist	Years since qualified	Years at Letaba Hospital
A	58	Male	Orthopaedics	26	35	8
B	56	Female	Paediatrics	27	31	12
C	45	Male	Dentistry	-	22	14
D	34	Male	Casualty MO	-	6	2
E	28	Female	Dentistry	-	4	3
F	52	Female	Paediatrics	17	31	3
G	49	Male	Internal medicine MO	-	25	7

MO, medical officer.

Four out of seven study participants had worked at Letaba Hospital for more than 5 years (see Table 1). This could have allowed them to form a critical view of FM, which contributed to the richness of the information. Participants with Cuban training and those who had been exposed to this discipline in other parts of the world (participants B, D and F) made a particularly positive contribution to this enquiry; in a sense they were able to evaluate the FM discipline from a different angle, looking at ideal standards.^22,23^

Eleven major themes on FM emerged from the individual interviews: (1) specialty status, (2) FM’s holistic approach, (3) FM focuses on prevention, (4) FM belongs to PHC, (5) FM is used to fill the gap, (6) FM is community-based, (7) FPs require specific skills, (8) FM has an important role in district hospitals, (9) FM is about continuity of care, (10) FM takes a leadership role in healthcare and (11) FM is at the centre of healthcare. Table 2 presents the themes and quotes from respondents.

**TABLE 2:** Themes with negative and positive perspectives.

Theme	Positive perceptions (quotes)	Negative perceptions (quotes)
1. Status of FM as a specialty	‘It’s [FM] just a specialty in its own right.’ (Participant C, 45 year old, Dentist)	‘They do not see it at this stage. I’m being honest, as a real specialty, they think it’s something halfway between a general practitioner and a specialist.’ (Participant A, 58 year old, Orthopedist)
	‘It [FM] is something that should be put in the front because it is about promoting a discipline; I think it is about simply raising awareness.’ (Participant C, 45 year old, Dentist)	-
2. FM has a holistic approach	‘I see them as people who will treat beyond the immediate presenting complaints … and they will assume or attempt a more holistic approach to helping that patient.’ (Participant C, 45 year old, Dentist)	‘They keep themselves busy with a lot of things … instead of pure, pure medical knowledge.’ (Participant A, 58 year old, Orthopedist)
3. FM belongs to PHC	‘My perception of FM is primary health first level of care.’ (Participant B, 56 year old, Paediatrician)	It’s disjointed … because there’s … it’s been emphasised as ‘casualty, casualty, casualty’ … and it’s not just casualty … you know. And I think that was part of the concerns of the HPCSA [Health Professions Council of South Africa] when they came to do a review recently.’ (Participant F, 52 year old, Paediatrician)
4. FPs require skills	‘Knowledge of every medicine. In other words, he may not be an orthopaedic surgeon, but he should give a level of care higher than what you would get from a regular GP. He doesn’t have to be an anaesthetist in the sense of the word. What he should have are skills.’ (Participant C, 45 year old, Dentist)	A great percentage of them, they cannot do it; after that MMed they still cannot do an appendectomy. Ordinary, basic surgery. I’m not even talking about exploration laparotomy or whatever.’ (Participant A, 58 year old, Orthopedist)
5. FM is community-based	‘If you want to win the war against disease, you need somebody that needs to go down to the clinic level, to interact with the community to find out what their diseases are.’ (Participant D, 34 year old, Medical officer Casualty)	Believe you me, once FM [*is*] done in this institution … I will emphasise it … if we take FM away, we will all be totally exposed in my unit.’ (Participant G, 58 year old, Orthopedist)
6. FM is about prevention	‘In South Africa, we are dedicated to cure, and not to prevent.’ (Participant B, 56 year old, Paediatrician)	-
7. FM is used to fill the gap	‘I perceive that FPs especially in this country use or avail themselves to [*close*] the gaps.’ (Participant C, 45 year old, Dentist)	-
	‘We will never have enough disciplines or enough specialists. So it doesn’t matter what you do, if you don’t look for alternatives to addressing these problems. So for me, it could be seen as a way to address those problems.’ (Participant C, 45 year old, Dentist)	
8. FM has an important role in district hospitals	‘You need more FPs out there to run those hospitals; and if you have good district hospitals, then your regional hospitals can then focus on what they are meant to focus on and actually provide regional care.’ (Participant F, 52 year old, Paediatrician)	-
9. FM is about continuity of care	‘They have to follow up. My understanding, as I worked in FM for many years, it’s one of the principles of the discipline.’ (Participant B, 56 year old, Paediatrician)	-
10. FM takes the lead in health-care	‘Personally I see FM as … [*it*] pertains to Letaba Hospital … that it is the mother of all other disciplines … in other words, if FM as a department is doing very well, it leads the other departments, and that is the way it has been. I got here about seven years ago.’ (Participant G, 49 year old, Medical Officer Internal medicine)	-
11. FM is the centre of healthcare	‘It is a specialty that is probably at the centre of health care.’ (Participant C, 45 year old, Dentist)	-

FM, family medicine; MMed, Master of Medicine; GP, general practitioner; FPs, family physicians.

**FIGURE 1:**
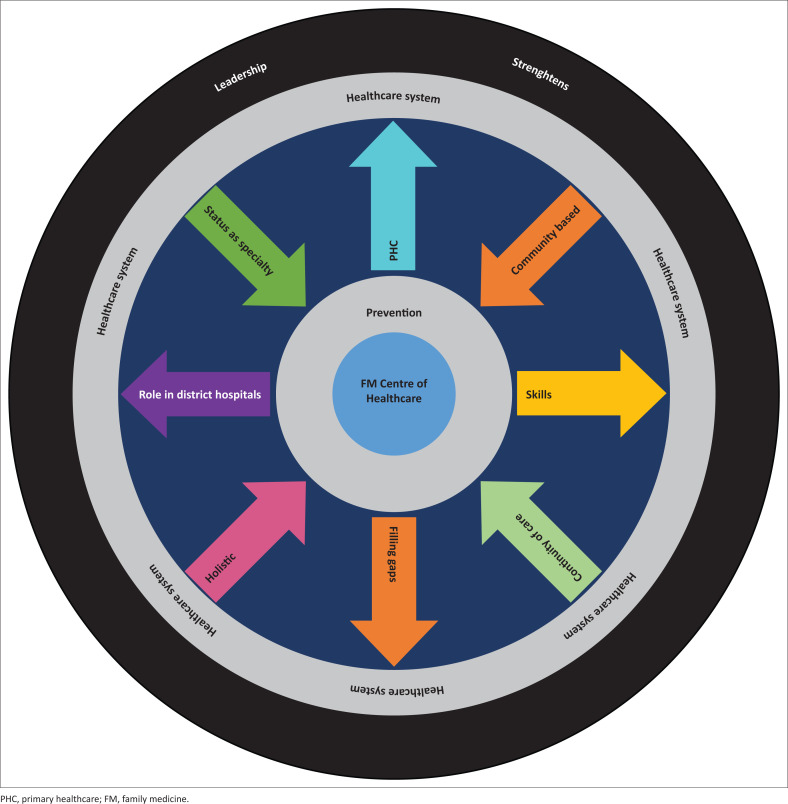
Model of family medicine as described by doctors at Letaba Hospital.

A ‘wheel’ (see Figure 1) depicts the 11 themes and their interrelatedness. Family medicine is placed at the centre of the health care system. Prevention is also featured at the centre as it forms the core of all FM’s activities. The other role descriptions of this discipline are represented by the spokes aimed in different directions. The outward spokes are activities that strengthen the healthcare system through the role of FM in PHC and district hospitals in filling the gaps and applying skills. The inward spokes (towards FM) are elements that would improve its functioning: being community-based, providing continuity of care, having a holistic approach, and the need of recognition of the discipline according to the responsibility it has currently. The outer layer of the wheel symbolises the major contribution of FM towards strengthening the healthcare system by means of its leadership role.

## Discussion

This was explorative qualitative research conducted at Letaba Hospital that aimed to explore the attitudes and perceptions of doctors about FM. It demonstrated that there are differences of opinion about FM as a specialty. Coming from medical colleagues, this is in contrast with the actual contribution of the discipline to the healthcare system that led to support from health dicision makers (leaders) globally.^10,24,25^ One participant had negative opinions about FM’s status as a specialty. He was convinced that it is not a specialty and placed the discipline somewhere between a specialty and ordinary general practice, whereas the rest of the participants had positive opinions of this discipline. One participant was of the view that there is poor knowledge about the subject globally, which he linked to the fact that FM is undermined by other disciplines. He therefore suggested that it should be upgraded and given names that ‘sell’, for example, ‘prevention’, ‘wellness’ or ‘social context of medicine’.

These views on FM are similar to the findings of Naidoo et al.,^24^ which showed that even prior to its establishment as a specialty, most general practitioners and specialists in SA were already in favour of it being recognised as such. The findings indicated that those who were not in favour of FM being recognised as a specialty had issues with competitiveness and remuneration. They also held misconceptions about the fact that they were already undertaking FM’s work and that such specialists would not be as qualified as specialists in other fields. Those respondents in Naidoo et al.’s study were not cognisant of the scope, extent and nature of the training of FM specialists. In a Canadian study, FM was reported to be subject to strong negative criticism from other specialties. The author proposed that:

Suggestions of potential solutions to gaining more respect included the need to create and develop relationships between family practitioners and other specialists and to support each other’s roles; to raise the profile of FM in universities and teaching hospitals; to change negative attitudes by promoting the expertise and role of FM to others; to demonstrate and maintain a comprehensive skills set; and to address intra-professional inequities and provide appropriate incentives.^16^

Our study has revealed that FM’s breadth makes it difficult for other disciplines to define its operational field. It was labelled as a ‘jack of all trades and master of none’ by one participant. Others thought that the concept is vague and that ‘nobody knows what is going on’. Ponka,^25^ Gibson^26^ and colleagues more recently in the Besrour Center’s work reported similar findings. This corroborates the findings of Du Toit and Couper^14^ as well as Voort et al.^15^ who reported a lack of awareness of FM, with many FPs finding themselves explaining their functions to both colleagues and the community.

The criticism in this study about the curative approach of medicine matches the observation of Flinkenflögel et al.^27^ that most African healthcare systems are hospital-based and depend on vertical programmes. For one participant who was very dedicated to outreach in district hospitals, there is a need for more FPs in district hospitals to ensure the facilities function properly. Regional hospitals could then focus on what they were originally intended to do. This same participant further noted that the FM training programme, based at the district hospital, has improved the quality of care in the relevant hospitals, yet the situation tended to deteriorate when registrars complete their training and leave. This supports the findings of Fletcher et al.^28^ that establishing a training site in the community for FM residency could result in better service delivery, improved teamwork and communication, and an improved working environment that would favour the retention and recruitment of FPs.

The Kenyan report by Voort et al.^15^ showed that FPs felt misused; instead of acting as the first specialist contact, FPs felt they were being told to fill ‘human resource gaps’, forcing them to take on the role of one of the other specialists and preventing them from carrying out their FM mandate as in our study. To rectify this situation, Ssenyonga proposed that FPs in healthcare systems in the developing world cannot be used as first line care providers, as is the case in the developed world, and being used merely as the next level of caregiver.^29^ Several authors have raised concerns about the excessive workload in family practice.^30,31,32,33^ This highlights the fact that although realities are different between the developed world and sub-Saharan countries, FM remains an overwhelmed medical field.

The matter pertaining to skills was understood differently by the participants. On the one hand, it was stressed that FPs must have better skills than ordinary general practitioners. The other group of participants underlined the fact that FPs do not have abilities superior to those of ordinary general practitioners; they felt that FPs lack basic surgical skills and therefore the curriculum in FM should allow registrars to spend more time in the surgical department to develop their surgical skills. One participant suggested that it would be more beneficial to have hands-on generalists than FPs with a MMed degree. This confirms the perception that the basic competence of FPs lies in ‘soft’ skills, such as counselling, and that they do not possess important clinical or surgical skills.^34^ However, surgical skills are important for FPs. The current postgraduate South African FM curriculum emphasises the importance of equipping future FPs with skills that are needed in a district hospital setting. The challenge with this curriculum is that future FPs could be better prepared for work in the district hospitals but underprepared for primary care.^34^ Of note in many settings, surgical skills beyond minor surgery are not expected of the FP.

Several participants felt that FM is an essential discipline in the healthcare system and in this study called it ‘the mother of all disciplines’ in praise of its leadership role. Mash^4^ describes a model of six key characteristics that define the job description of an FP in SA: (1) he/she is a care provider, (2) a consultant who gives support to primary health, (3) a capacity-builder who is involved in clinical training and mentoring of healthcare staff, (4) a supervisor of registrars and undergraduates, (5) a manager and (6) a community leader. Recent studies by Von Pressentin^10,11,12,13^ have clearly emphasised the impact of the FP in the districts of SA and even the need to deploy far more of them as a crucial solution to the ailing primary health system.

## Recommendations

We recommend the following:

- Focus group discussions could have yielded different outcomes and would have helped to improve triangulation.
- Future studies using focus group discussions are recommended.
- Strategies need to be developed to create a positive attitude towards FM by other disciplines.
- The image and understanding of FM needs to be improved, and negative perceptions corrected.
- A study considering allied medical professionals should be conducted.

## Limitations

The findings of this study cannot be generalised as they are founded on a qualitative inquiry at one hospital, with a small number of participants. Data may be outdated as it was collected in 2013; the perceptions of participants may have changed with time in the local context.

Nevertheless, the results are transferable to similar contexts. As a purposeful sample of participants, not all departments were represented in this study. Representatives from departments that were omitted might have presented new ideas and insights. During data collection there was a failure to create focus group interviews that could have enriched the discussions of the individual interviews. Reflexivity is a common confounding issue in qualitative research.

Reflexivity relates to sensitivity regarding the ways in which the researcher and the research process may shape the data obtained, including the purpose of prior assumptions and experience.^35^ Prior to the research, the researcher’s assumption was that doctors at Letaba Hospital did not understand FM, Surprisingly, they understood FM better than anticipated. It is possible that, since all the doctors knew the principal researcher as a family practitioner, they would likely abstain from criticism and wanted to provide answers showing a positive perception and good understanding of the FM discipline. However, the researchers tried to reduce this by using an independent outside professional interviewer, with confidentiality assured. In addition, the positive perceptions of FM could also have been influenced by the richness of data obtained from doctors from Cuba who have been more exposed to FM; whereas FM doctors themselves were excluded from the study. The model established from this study could have been influenced by the fact that the main researcher and the research supervisor are from FM, having himself a positive perception of the discipline. Triangulation in each step of analysis and results could minimise that influence. Nonetheless, more time for reflexivity could have generated a different model.

## Conclusion

The whole image of FM represented as a wheel model in this research painted a more positive picture of the 11 aspects that define the discipline. There were only a few negative perceptions of the discipline, mostly involving issues relating to the scope of the discipline of FM and the status of the subject as a specialty. Although many concerns were raised regarding the curative hospital-based system that currently dominates the healthcare system of SA, the overwhelming feeling was that FM should be afforded the opportunity to excel in the sphere of preventative medicine; most of the participants believe this is where the strengths of FM lie. Strategies need to be established that can create a positive attitude towards FM in certain disciplines.
